# High Liver Enzyme Concentrations are Associated with Higher Glycemia, but not with Glycemic Variability, in Individuals without Diabetes Mellitus

**DOI:** 10.3389/fendo.2017.00236

**Published:** 2017-09-13

**Authors:** Raymond Noordam, Debbie Vermond, Hermijntje Drenth, Carolien A. Wijman, Abimbola A. Akintola, Sabrina van der Kroef, Steffy W. M. Jansen, Neline C. Huurman, Bianca A. M. Schutte, Marian Beekman, P. Eline Slagboom, Simon P. Mooijaart, Diana van Heemst

**Affiliations:** ^1^Department of Internal Medicine, Section of Gerontology and Geriatrics, Leiden University Medical Center, Leiden, Netherlands; ^2^Leyden Academy on Vitality and Ageing, Leiden, Netherlands; ^3^Department of Medical Statistics and Bioinformatics, Section Molecular Epidemiology, Leiden University Medical Center, Leiden, Netherlands; ^4^Institute for Evidence-Based Medicine in Old Age, IEMO, Leiden, Netherlands

**Keywords:** continuous glucose monitoring, liver enzymes, glycemia, glycemic variability, cross-sectional cohort study

## Abstract

**Background:**

Elevated concentrations of liver enzymes have been associated with an increased risk of developing type 2 diabetes mellitus. However, it remains unclear to which specific aspects of diurnal glucose metabolism these associate most. We aimed to investigate the associations between liver enzyme concentrations and 24 h-glucose trajectories in individuals without diabetes mellitus from three independent cohorts.

**Methods:**

This cross-sectional study included 436 participants without diabetes mellitus from the Active and Healthy Aging Study, the Switchbox Study, and the Growing Old Together Study. Fasting blood samples were drawn to measure gamma-glutamyltransferase (GGT), alanine transaminase, and aspartate transaminase. Measures of glycemia (e.g., nocturnal and diurnal mean glucose levels) and glycemic variability (e.g., mean amplitude of glucose excursions) were derived from continuous glucose monitoring. Analyses were performed separately for the three cohorts; derived estimates were additionally meta-analyzed.

**Results:**

After meta-analyses of the three cohorts, elevated liver enzyme concentrations, and specifically elevated GGT concentrations, were associated with higher glycemia. More specific, participants in the highest GGT tertile (GGT ≥37.9 U/L) had a 0.39 mmol/L (95% confidence interval: 0.23, 0.56) higher mean nocturnal glucose (3:00 to 6:00 a.m.) and a 0.23 mmol/L (0.10, 0.36) higher diurnal glucose (6:00 to 0:00 a.m.) than participants in the lowest GGT tertile (GGT <21.23 U/L). However, elevated liver enzyme concentrations were not associated with a higher glycemic variability.

**Conclusion:**

Though elevated liver enzyme concentrations did not associate with higher glycemic variability in participants without diabetes mellitus, specifically, elevated GGT concentrations associated with higher glycemia.

## Introduction

It has been well recognized that the prevalence of type 2 diabetes mellitus (T2DM) is increasing worldwide (1, 2). In literature, studies have focused on the identification of risk factors associated with a higher risk of developing T2DM, in order to identify potential targets for (therapeutic) interventions and to understand the different pathophysiological mechanisms. Several risk factors have been identified for T2DM, which include both inherited factors (e.g., genetic factors) (3) and modifiable risk factors such as adiposity and high caloric intake (4). In addition, disease conditions as non-alcoholic fatty liver disease (NAFLD) or a preclinical higher degree of liver adiposity have also been described to increase the risk of T2DM (5–7). Both NAFLD and increased liver adiposity are reflected by elevated blood concentrations of liver enzymes [notably alanine transaminase (ALT), aspartate transaminase (AST), and gamma-glutamyltransferase (GGT)]. Elevations of blood concentrations of liver enzymes have been repeatedly associated with an increased risk of developing T2DM in multiple settings (8–11). Nevertheless, there are still limited data available on the association between elevated liver enzyme concentrations and the dynamic aspects of glucose metabolism over 24 h, like glycemia and glycemic variability. Such insights will provide additional information about the pathophysiological mechanisms in which elevated liver enzyme concentrations are involved.

Measures of glycemia and glycemic variability, as derived from 24 h glucose trajectories, can be obtained with continuous glucose monitoring (CGM), which uses a minimally invasive device to measures glucose concentrations for a period up to 7 days, while the participants pursue their regular daily life activities (12–14). Using this device, high concentrations of serum ALT have previously been associated with higher glycemia (specifically glycemia during the nocturnal period of the 24 h period) in 322 Chinese individuals without diabetes mellitus (13). However, this study did not investigate the other liver enzymes AST and GGT, nor did it study glycemic variability, and generalization to Western populations has yet to be determined.

To provide further insights in the association between liver enzyme concentrations and 24 h glucose trajectories, we examined the associations between liver enzyme (ALT, AST, and GGT) and CGM-derived measures of glycemia and glycemic variability over 3 days in three independent populations of middle-aged individuals without diabetes mellitus.

## Materials and Methods

### Study Settings

The present study was conducted using data of the Active and Healthy Aging (AGO) Study, the Switchbox Study, and the Growing Old Together Study (GOTO). The designs and recruitment strategies of the three studies have been described in more detail elsewhere (15–18). The AGO, Switchbox, and GOTO studies have been approved by the medical ethics committee of the Leiden University Medical Center, Leiden, the Netherlands. Written informed consent was obtained from all study participants.

#### The Active and Healthy Aging (“Actief en Gezond Oud”; AGO)

The AGO study aimed to investigate the effect of a web-based lifestyle intervention program, with the intention to increase physical activity, on metabolic health. For this study, individuals aged 60–70 years living in the city of Leiden, the Netherlands, were recruited. Individuals with a history of diabetes mellitus, an active lifestyle (more than 3 h of physical exercise or cycling per week), or a contraindication to increase physical activity were not included. In total, 243 individuals were enrolled and were randomized for either the intervention program or the control arm of the AGO study. The AGO study was registered at the Dutch Trial Register (http://www.trialregister.nl) as NTR3045.

#### The Switchbox Study

The Switchbox Study aimed to investigate the biological mechanisms underlying familial longevity. Individuals were enrolled from the ongoing, and larger, Leiden Longevity Study (19). Participants were eligible when their age was between 55 and 77 years and they had a stable body mass index (BMI) between 19 and 33 kg/m^2^. Participants were not eligible for participation in the Switchbox study if they had a fasting glucose above 7 mmol/L, if they had significant chronic, renal, hepatic, or endocrine disease, or if they used any medication known to influence lipolysis, thyroid function, glucose metabolism, GH/IGF-1 secretion, or any other hormonal axis. Moreover, participants were excluded if they had a recent trans meridian flight, smoking addition, use of more than 20 U of alcohol, and extreme diet therapies. In total, 135 individuals were enrolled in the Switchbox study.

#### The Growing Old Together (GOTO) Study

The GOTO study aimed to investigate the effect of a combined physical activity and diet intervention on metabolic and metabolomic phenotypes. Similar to the Switchbox Study, participants were enrolled from the Leiden Longevity Study (19). Individuals of ages between 46 and 75 years and with a BMI between 23 and 35 kg/m^2^ were eligible to participate. Exclusion criteria were: treated for diabetes mellitus, a fasting glucose level above 7 mmol/L, a weight change of more than 3 kg during the last 6 months, engagement in heavy/intensive physical activity (top sport of physically heavy work), any disease or condition that seriously affects body weight (e.g., cancer, heart failure, COPD), recent immobilization for >1 week, psychiatric or behavioral problems, use of thyroid mediation or immunosuppressive drugs, concurrent participation in any other intervention study or weight management program, or not having a general practitioner. In total, 163 individuals were enrolled in the GOTO study. The GOTO study was registered at the Dutch Trial Register (http://www.trialregister.nl) as NTR3499.

### Study Design and Population

The present study was conducted in a cross-sectional setting in participants without T2DM. As AGO and GOTO were both intervention studies, we used only the baseline data (prior to the intervention) for the present study. In AGO, we identified two participants with a potential newly diagnosed T2DM and were, therefore, excluded from the present study. After exclusion of participants with missing data on either CGM or liver enzyme concentrations (*N* = 15 in AGO; *N* = 19 in Switchbox), and excluding participants in GOTO who already participated in the Switchbox study, our total study population contained 436 participants with complete data (*N* = 226 from AGO, *N* = 116 from Switchbox, *N* = 94 from GOTO).

### Biochemical Analyses

After an overnight fast, blood samples were drawn from all study participants. All measurements were performed with fully automated equipment from Roche Diagnostics (Almere, the Netherlands). ALT, AST, and GGT levels were determined using the Abbott ci8200. ALT and AST were measured using the NADH (with P-5′-P) methodology and GGT was measured using the substrate l-Gamma-Glutamyl-3-carboxy-4-nitroanilide methodology. All measurements were performed at the Department of Clinical Chemistry and Laboratory Medicine, Leiden University Medical Center, Leiden, the Netherlands.

### Anthropometrics

Weight and height were measured at the study center by research nurses. BMI was calculated by dividing the weight (in kilograms) by height (in meters) squared. Waist-to-hip ratio was calculated by dividing waist circumference by hip circumference, which were measured at the study center. Percentage of body fat was determined according to a mobile Bioelectrical Impedance Analysis system (Bodystat^®^ 1500 Ltd., Isle of Man, British Isle).

### Glucose Measurements

In the three studies, CGM was performed with the Mini-Med^®^ CGM system (Medtronic Minimed Inc., Northridge, CA, USA). For five consecutive days, interstitial glucose levels were monitored with a glucose sensor (Sof-Sensor^®^, Medtronic, Minimed Inc., Northridge, CA, USA) inserted into the subcutaneous abdominal fat tissue. For calibration of the sensor, participants measured their capillary blood glucose four times a day by means of a finger prick. The participants were supported to continue their normal daily activities. Moreover, they were asked to register their food intake, medication, and physical exercise during the study. In line with the guidelines from the manufacturer, the first and fifth day of the measurement were excluded, as these were considered least accurate, leaving 3 days (covering 72 h) of data for the present study.

On the basis of the retrieved glucose trajectories, we calculated multiple measures of glycemia and glycemic variability for each participant separately. As measures of glycemia, we calculated the 72-h mean glucose concentration, the mean diurnal glucose concentration (6:00 to 0:00 a.m.), and the mean nocturnal glucose level (3:00 to 6:00 a.m.; a period when all participants were considered to be sleeping). As measures of glycemic variability, we calculated the 72-h SD mean amplitude of glucose excursion (MAGE), and the mean of daily difference (MODD). The MAGE determines intraday glycemic variability (20), whereas the MODD determines interday variability (21). These calculations for glycemia and glycemic variability have been validated in non-diabetic individuals before (14), and have already been used in previous studies (12, 22).

### Statistical Analyses

Characteristics of the study populations were presented separately for the AGO, Switchbox, and GOTO study populations as mean (SD), number of cases (percentage), or median (interquartile range; non-normally distributed data only).

We divided the three study populations in three groups based on their blood liver enzyme concentration, because it has previously been shown that the association between elevated GGT and T2DM was not linear (10). Mean liver enzyme concentrations as well as the variation in liver enzyme concentrations between the three cohorts were observed to be considerably different. To obtain tertiles that were comparable between the cohorts in terms of mean liver enzyme concentrations, we divided the participants from AGO (as being the largest cohort contributing to the present study) in tertiles based on their blood liver enzyme concentrations and applied the boundaries of these strata to the other two study populations. By using the same boundaries for all groups, it was possible to compare and to subsequently meta-analyze the results of the three study populations.

All statistical analyses assessing the associations between the blood liver enzyme concentrations and CGM-derived measures of glycemia and glycemic variability were conducted using multivariable linear regression analyses with the strata of the liver enzyme concentrations as independent variables and the measures of glycemia and glycemic variability as dependent variables. Analyses were done separately for the AGO, Switchbox, and GOTO study populations. For the analyses, we considered participants in the group with the lowest blood liver enzyme concentration as the reference group. Analyses were adjusted for age, sex, and BMI using STATA v12.0 (StataCorp LP, College Station, TX, USA). In sensitivity analyses, we adjusted the analyses on the measures of glycemic variability for the 72-h mean glucose concentration. Results of the study populations were combined using a fixed effect inverse-variance weighted meta-analysis as implemented in the rmeta package for the R statistical environment (23). All results were presented as mean difference with respect to the reference group and with a 95% confidence interval (CI). A two-sided *p*-value < 0.05 was considered statistically significant.

## Results

### Characteristics of the Three Study Populations

For the present study, we included 226 participants from the AGO study, 116 participants from the Switchbox study, and 94 participants from the GOTO study (Table 1). The three independent cohorts had a similar mean age (±65 years). Specifically participants from the AGO study had a higher percentage of men (59.7 versus 48.3 and 52.1%), had a higher mean BMI (28.9 versus 26.0 and 26.8 kg/m^2^), and had a higher percentage of body fat (36.2 versus 32.3 and 29.4%) compared with the Switchbox and GOTO studies. In addition, participants from AGO had a higher mean GGT (27.3 versus 23.5 and 20.2 U/L), a higher mean fasting glucose (5.7 versus 5.4 and 5.0 mmol/L), and a higher median fasting insulin (11.2 versus 6.0 and 8.6 mU/L) compared with the Switchbox and GOTO studies.

**Table 1:** Characteristics of the study population.

	AGO (*N* = 226)	Switchbox (*N* = 116)	GOTO (*N* = 94)
**Demographics**							
Age (years), mean (SD)	64.8 (2.9)	65.7 (6.2)	63.4 (5.4)
Men, *n* (%)	135 (59.7)	56 (48.3)	49 (52.1)
**Body composition**							
Body mass index (kg/m^2^), mean (SD)	28.9 (4.5)	26.0 (4.1)	26.8 (2.3)
Fat percentage, mean (SD)^a^	36.2 (7.8)	32.3 (8.2)	29.4 (8.2)
**Blood measurements**							
Aspartate aminotransaminase (U/L), median (IQR)	24.7 (22.1, 29.1)	24.0 (21.0, 28.0)	23.9 (21.6, 27.5)
Alanine aminotransaminase (U/L), median (IQR)	18.2 (14.1, 25.3)	22.0 (19.0, 26.0)	15.3 (12.7, 17.8)
Gamma-glutamyltransferase (U/L), median (IQR)	27.3 (18.9, 45.1)	23.5 (15.0, 33.8)	20.2 (14.9, 30.2)
Blood glucose (mmol/L), mean (SD)	5.7 (0.7)	5.4 (0.5)	5.0 (0.5)
Insulin (mU/L), median (IQR)	11.2 (7.6, 16.7)	6.0 (3.0, 8.2)	8.6 (5.7, 11.1)
**Measurements derived with continuous glucose monitoring**							
72-h mean glucose (mmol/L)	5.51 (0.62)	5.29 (0.51)	5.21 (0.51)
Nocturnal glucose (mmol/L)	4.98 (0.72)	4.58 (0.69)	4.42 (0.57)
Diurnal glucose (mmol/L)	5.70 (0.65)	5.47 (0.51)	5.42 (0.54)
MAGE	2.32 (0.84)	2.21 (0.73)	2.26 (0.63)
72-h SD	0.97 (0.31)	0.94 (0.24)	0.93 (0.27)
MODD	0.87 (0.28)	0.89 (0.26)	0.83 (0.24)

*AGO, “Actief en Gezond Oud”; IQR, interquartile range; MAGE, mean amplitude of glycemic excursions; MODD, mean of daily differences*.

*^a^Missing for two participants in the AGO Study*.

### Associations between Liver Enzyme Concentrations and CGM-Derived Measures of Glycemia

**Figure 1:**
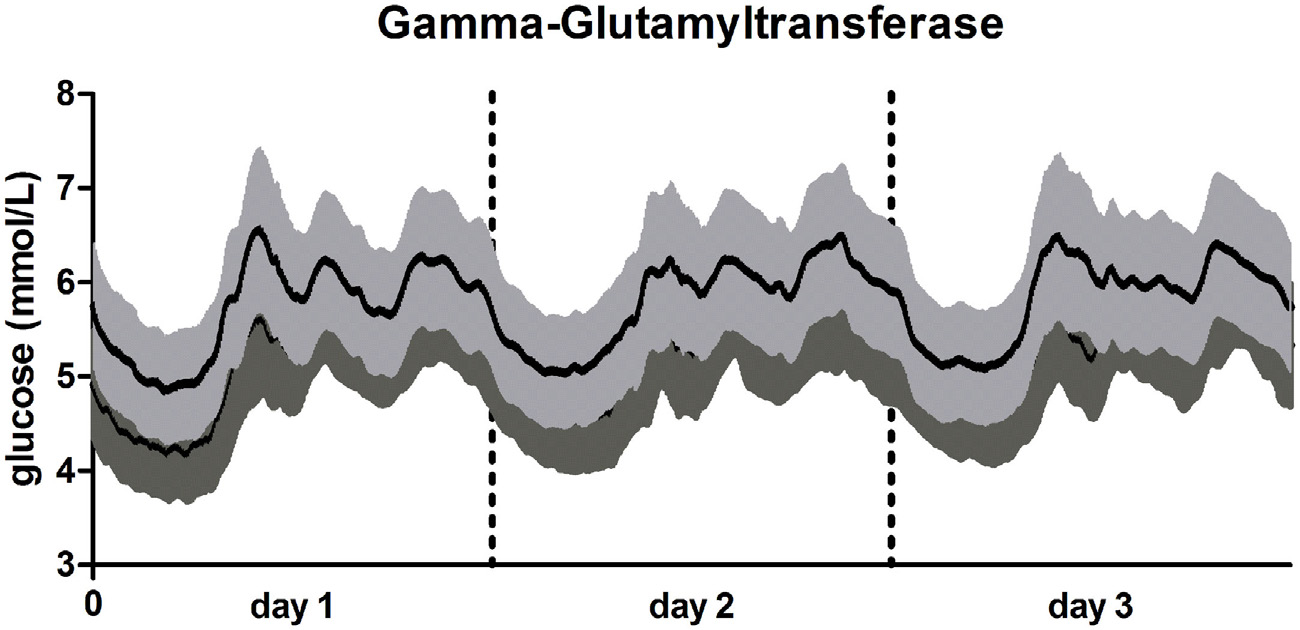
72-h glucose trajectories in participants with low and high gamma-glutamyltransferase (GGT). Line in light gray: participants with high GGT (≥37.9 U/L; *N* = 111). Line in dark gray: participants with low high GGT (<21.23 U/L; *N* = 179). Data presented as mean glucose concentration with SE for every 5 min over a 72 h period.

A graphical visualization of the mean glucose trajectories over 72 h of participants in the group of the lowest and highest GGT concentration is presented in Figure 1.

After meta-analyzing the results of the three studies (Table 2), participants allocated to the group with the highest GGT concentration (GGT ≥37.90 U/L) had a 0.27 mmol/L (95% CI: 0.14, 0.40) higher 72-h mean glucose concentration, a 0.39 mmol/L (95% CI: 0.23, 0.56) higher mean nocturnal glucose concentration, and a 0.23 mmol/L (95% CI: 0.10, 0.36) higher diurnal glucose concentration, compared with participants allocated to the group with the lowest GGT blood concentration (GGT < 21.23 U/L). Similar results were observed in the three individual studies (Table S1 in Supplementary Material), although sometimes with large confidence intervals due to the small number of participants in some of the subgroups.

**Table 2:** Associations of liver enzyme concentrations and measures of glycemia in the meta-analyses.

	GGT		ALT		AST	
	*N*	Beta (95% CI)	*N*	Beta (95% CI)	*N*	Beta (95% CI)
**72-h mean glucose (mmol/L)**													
Low	179	0 (ref)	136	0 (ref)	167	0 (ref)
Medium	146	0.15 (0.03, 0.27)*	164	0.13 (0.00, 0.25)*	138	−0.07 (−0.13, −0.01)
High	111	0.27 (0.14, 0.40)*	139	0.21 (0.01, 0.42)*	134	−0.07 (−0.19, 0.05)
**Nocturnal glucose (mmol/L)**													
Low	179	0 (ref)	136	0 (ref)	167	0 (ref)
Medium	146	0.16 (0.01, 0.32)*	164	0.13 (−0.02, 0.28)	138	−0.03 (−0.19, 0.13)
High	111	0.39 (0.23, 0.56)*	139	0.25 (0.07, 0.42)*	134	−0.09 (−0.23, 0.06)
**Diurnal glucose (mmol/L)**																				
Low	179	0 (ref)	136	0 (ref)	167	0 (ref)
Medium	146	0.13 (0.01, 0.26)*	164	0.12 (−0.01, 0.25)	138	−0.08 (−0.21, 0.05)
High	111	0.23 (0.10, 0.36)*	139	0.20 (0.05, 0.35)*	134	−0.06 (−0.19, 0.07)

*ALT, alanine transaminase; AST, aspartate transaminase; CI, confidence interval; GGT, gamma-glutamyltransferase; *N*, number of participants in stratum (all three cohorts combined)*.

Analyses adjusted for age, sex, and body mass index. Analyses in Switchbox and GOTO additionally corrected for familial relationships. Ranges GGT: low, <21.23 U/L; medium, 21.23–37.89 U/L; high, ≥37.90 U/L. Ranges ALT: low, <15.4 U/L; medium, 15.4–22.39; high, ≥22.4 U/L. Ranges AST: low, <23.30 U/L; medium, 23.3–27.29 U/L; high, ≥27.3 U/L. Data presented as the mean difference in outcome (with 95% confidence interval) with respect to the reference group; *p-value < 0.05.

Similar results, although with somewhat smaller effect sizes than with GGT (especially for the association with nocturnal glucose), were observed with ALT (Table 2). For example, participants allocated to the group with the highest ALT blood concentrations (ALT ≥22.40 U/L) had a 0.21 (95% CI: 0.07, 0.20) mmol/L higher 72-h mean glucose concentration than participants in the group with the lowest ALT blood concentrations (ALT <15.40 U/L). Again, these observations were similarly observed in the three study populations separately (Table S2 in Supplementary Material). We found no evidence, also not after meta-analyses of the three study populations, that high AST blood concentrations were associated with any of the investigated CGM-derived measures of glycemia (Table 2) neither in one of the three individual cohorts (Table S3 in Supplementary Material).

### Associations between Liver Enzyme Concentrations and CGM-Derived Measures of Glycemic Variability

After meta-analyses of the results of the three study populations (Table 3), we found no evidence that participants in the group with the highest GGT blood concentration (GGT ≥ 37.90 U/L) had, compared to participants in the group with the lowest GGT blood concentration (GGT < 21.23 U/L), a higher MAGE (difference: 0.04; 95% CI: −0.11, 0.18), a higher 72-h SD (difference: −0.03; 95% CI: −0.13, 0.02), or a higher MODD (difference: 0.01; 95% CI: −0.07, 0.08). Similar results were obtained for ALT and AST (Table 3), as well as for the three individual studies (Tables S4–S6 in Supplementary Material). The results did not materially change when we additionally adjusted for 72-h mean glucose concentration (results not shown).

**Table 3:** Association gamma-glutamyltransferase and measures of glycemic variability.

	GGT		ALT		AST	
	*N*	Beta (95% CI)	*N*	Beta (95% CI)	*N*	Beta (95% CI)
**MAGE**													
Low	179	0 (ref)	136	0 (ref)	167	0 (ref)
Medium	146	0.12 (−0.05, 0.29)	164	−0.05 (−0.23. 0.14)	138	0.00 (−0.10, 0.09)
High	111	0.04 (−0.11, 0.18)	139	−0.10 (−0.31, 0.10)	134	−0.07 (−0.17, 0.03)
**72-h SD**													
Low	179	0 (ref)	136	0 (ref)	167	0 (ref)
Medium	146	0.04 (−0.03, 0.10)	164	−0.04 (−0.10, 0.03)	138	−0.07 (−0.13, −0.01)
High	111	−0.03 (−0.10, 0.04)	139	−0.05 (−0.13, 0.02)	134	−0.03 (−0.10, 0.04)
**MODD**																				
Low	179	0 (ref)	136	0 (ref)	167	0 (ref)
Medium	146	0.05 (−0.01, 0.11)	164	−0.03 (−0.10, 0.03)	138	−0.06 (−0.12, 0.00)
High	111	0.01 (−0.07, 0.08)	139	−0.04 (−0.11, 0.03)	134	−0.04 (−0.10, 0.03)

*ALT, alanine transaminase; AST, aspartate transaminase; CI, confidence interval; GGT, gamma-glutamyltrasnferase; MAGE, mean amplitude of glycemic excursions; MODD, mean of daily differences; *N*, number of participants in stratum (all three cohorts combined)*.

*Analyses adjusted for age, sex, and body mass index. Analyses in Switchbox and GOTO additionally corrected for familial relationships. Ranges GGT: low, <21.23 U/L; medium, 21.23–37.89 U/L; high, ≥37.90 U/L. Ranges ALT: low, <15.4 U/L; medium, 15.4–22.39; high, ≥22.4 U/L. Ranges AST: low, <23.30 U/L; medium, 23.3–27.29 U/L; high, ≥27.3 U/L. Data presented as difference in outcome (with 95% confidence interval) with respect to the reference group*.

## Discussion

Within the present study, we aimed to investigate the associations between liver enzyme concentrations and measures of glycemia and glycemic variability in a cross-sectional study comprising middle-aged individuals without T2DM. The results of the present study provided evidence that, using data from three independent studies, elevated liver enzyme concentrations, and specifically elevated GGT concentrations, were associated with higher glycemia during day and night. However, the present study did not provide evidence that elevated liver enzyme concentrations were associated with higher glycemic variability.

The results of the associations between elevated liver enzyme concentrations and increased CGM-derived measures of glycemia are in line with previous studies on the association between elevated liver enzyme concentrations and fasting glucose concentrations (24–27). Our study showed strong associations between elevated GGT concentrations and measures of glycemia. In line, there is strong consensus that elevated blood GGT concentrations are associated with an increased risk to develop T2DM (10). The results of the present study indicate that the previously observed association between GGT and elevated fasting blood glucose concentrations persists over the day, which was reflected in our analyses on the mean nocturnal, as well as on the mean diurnal glucose concentrations. This might indicate that the biological mechanism behind the association between elevated liver enzyme concentrations and measures of glycemia involves a decreased glucose disposal.

The three investigated liver enzyme concentrations have been shown to be well correlated with each other in previous studies (27). However, the present study also found associations between elevated ALT concentrations and measures of glycemia, but no associations were found between elevated AST concentrations and measures of glycemia. Nevertheless, the associations for AST were generally less strong than with GGT although the results with ALT also reached the level of statistical significance. In line, there are a number of publications that showed that elevated ALT concentrations are associated with increased fasting blood glucose concentrations (13, 26–28). For example, in a Chinese-ancestry population, an elevated blood ALT concentration was associated with an increased fasting blood glucose concentration, but this effect was only observed in women (26). The only previous study using CGM-derived measures of glycemia observed associations between ALT and mean nocturnal and diurnal glucose concentrations (13). Nevertheless, a study conducted in participants of Taiwanese ancestry observed an association between ALT and metabolic syndrome, but not with elevated fasting glucose concentrations and T2DM (29). Together with our results, this supports that the investigated liver enzymes reflect different aspects of liver function. This indicates that blood ALT and AST concentrations are less related to glucose metabolism than blood GGT concentrations.

The present study is the first to examine the associations between liver enzyme concentrations and measures of glycemic variability. A high glycemic variability is a generally known risk factor for micro- and macrovascular complications in patients diagnosed with T2DM (30). However, we found that elevated liver enzyme concentrations were not associated with a higher glycemic variability. The lack of an association with glycemic variability has previously also been observed for genetic variation in the *TCF7L2* gene (31), one of the strongest genetic risk factors for T2DM. This might indicate that glycemia rather than glycemic variability is the most important risk factor for the development of T2DM. Therefore, the importance (if any) of glycemic variability in populations of individuals without diabetes mellitus needs to be further elucidated.

The results of the present study suggest that increased liver enzyme concentrations are associated with decreased glucose disposal. In the literature, there is currently debate on whether the association between GGT and increased glycemia and T2DM are causal (8, 32, 33). The observations done in the present study could reflect a common cause, rather than a causal relation. Several mechanisms have been proposed to underpin the observed associations between GGT and risk of T2DM (34, 35). As a potential mechanism explaining the results of the present study, we hypothesize that liver fat content might be the common cause of elevated blood GGT concentrations and elevated levels of glycemia. Liver fat content was shown to correlate with blood GGT concentrations (36), as well as to associate with several established risk factors for T2DM, including obesity, oxidative stress, and inflammation. However, additional studies are required to further elucidate on this hypothesis.

The present study has a number strengths and limitations. The main strength of the present study is that data on glucose levels were collected every 5 min over a 72-h period during which participants were asked to continue their normal daily life activities. This provided the opportunity for a detailed analysis of nocturnal and diurnal patterns, but also of the variation in measurements. Furthermore, the present study was conducted using a large sample size in which CGM data have been collected. Also, the present study was conducted using data collected from three independent study populations with different inclusion criteria and population characteristics. Notably, the AGO study population was recruited with the intention to improve lifestyle (16), and using different in- and exclusion criteria, participants from Switchbox and GOTO were enrolled from the Leiden Longevity Study, which included participants based on their propensity to become long-lived together with their partners as controls (15). As the results were similar in the three study populations, this emphasizes the robustness of our findings across populations with different characteristics. Nevertheless, because of the observational nature of the study, no causality was ascertained and results might have been harmed by residual confounding and/or reverse causality. However, since observations were similar in three distinct populations with different study characteristics, the effect of residual confounding in the analyses was considered minimal.

In summary, the results of the present study from individuals without diabetes mellitus indicate that elevated liver enzyme concentrations, specifically elevated GGT blood concentrations, are associated with increased glycemia, but not with increased glycemic variability.

## Ethics Statement

The three cohorts described in the present study have been approved by the medical ethics committee of the Leiden University Medical Center, Leiden, the Netherlands.

## Author Contributions

Study design (RN, DV, HD, PS, SM, DH); acquisition of data (CW, AA, SK, NH, SJ, BS, MB); data analyses (RN, DV, HD); drafted the manuscript (RN, DV, HD, DH); critically commented and final approval of the manuscript (RN, DV, HD, CW, AA, SK, NH, SJ, BS, MB, PS, SM, DH).

## Conflict of Interest Statement

The authors declare that the research was conducted in the absence of any commercial or financial relationships that could be construed as a potential conflict of interest. The reviewer, LB, and the handling editor declared their shared affiliation.
