# Supplementary material for: High Liver Enzyme Concentrations are Associated with Higher Glycemia, but not with Glycemic Variability, in Individuals without Diabetes Mellitus
Source: Front Endocrinol (Lausanne). 2017 Sep 13;8:236. doi: 10.3389/fendo.2017.00236 (PMC5601417; doi:10.3389/fendo.2017.00236)
Supplement: Supplementary file 1 [file table_1.docx]

| **Table A.1:** Association gamma-glutamyltransferase and measures of glycaemia in three different cohorts | | | | | | | | | | |
| --- | --- | --- | --- | --- | --- | --- | --- | --- | --- | --- |
|  | Range |  | AGO | |  | Switchbox | |  | GOTO | |
|  | U/L |  | N | Beta (95% CI) |  | N | Beta (95% CI) |  | N | Beta (95% CI) |
| **24-h mean glucose (mmol/L)** |  |  |  |  |  |  |  |  |  |  |
| Low | <21.23 |  | 74 | 0 (ref) |  | 54 | 0 (ref) |  | 51 | 0 (ref) |
| Medium | 21.23 – 37.89 |  | 77 | 0.17 (-0.02; 0.36) |  | 41 | 0.17 (-0.04; 0.38) |  | 28 | 0.08 (-0.16; 0.33) |
| High | >37.90 |  | 75 | 0.34 (0.15; 0.54) |  | 21 | 0.08 (-0.16; 0.31) |  | 15 | 0.38 (0.11; 0.66) |
|  |  |  |  |  |  |  |  |  |  |  |
| **Noctornal glucose (mmol/L)** |  |  |  |  |  |  |  |  |  |  |
| Low | <21.23 |  | 74 | 0 (ref) |  | 54 | 0 (ref) |  | 51 | 0 (ref) |
| Medium | 21.23 – 37.89 |  | 77 | 0.26 (0.03; 0.50) |  | 41 | 0.16 (-0.14; 0.46) |  | 28 | 0.03 (-0.24; 0.31) |
| High | >37.90 |  | 75 | 0.42 (0.18; 0.66) |  | 21 | 0.08 (-0.29; 0.44) |  | 15 | 0.57 (0.26; 0.87) |
|  |  |  |  |  |  |  |  |  |  |  |
| **Diurnal glucose (mmol/L)** |  |  |  |  |  |  |  |  |  |  |
| Low | <21.23 |  | 74 | 0 (ref) |  | 54 | 0 (ref) |  | 51 | 0 (ref) |
| Medium | 21.23 – 37.89 |  | 77 | 0.11 (-0.09; 0.31) |  | 41 | 0.18 (-0.03; 0.38) |  | 28 | 0.10 (-0.15; 0.36) |
| High | >37.90 |  | 75 | 0.31 (0.10; 0.51) |  | 21 | 0.08 (-0.14; 0.30) |  | 15 | 0.33 (0.04; 0.63) |

Analyses adjusted for age, sex, and body mass index. Data presented as the mean difference in outcome (with 95% confidence interval) with respect to the reference group.

| **Table A.2:** Association alanine-aminotransferase and measures of glycaemia in three different cohorts | | | | | | | | | | |
| --- | --- | --- | --- | --- | --- | --- | --- | --- | --- | --- |
|  | Range |  | AGO | |  | Switchbox | |  | GOTO | |
|  | U/L |  | N | Beta (95% CI) |  | N | Beta (95% CI) |  | N | Beta (95% CI) |
| **24-h mean glucose (mmol/L)** |  |  |  |  |  |  |  |  |  |  |
| Low | <15.40 |  | 73 | 0 (ref) |  | 12 | 0 (ref) |  | 51 | 0 (ref) |
| Medium | 15.40 – 22.39 |  | 79 | 0.09 (-0.10; 0.27) |  | 53 | 0.29 (0.01; 0.56) |  | 32 | 0.08 (-0.12; 0.29) |
| High | ≥22.40 |  | 77 | 0.18 (-0.01; 0.37) |  | 51 | 0.41 (0.13; 0.68) |  | 11 | -0.01 (-0.38; 0.37) |
|  |  |  |  |  |  |  |  |  |  |  |
| **Noctornal glucose (mmol/L)** |  |  |  |  |  |  |  |  |  |  |
| Low | <15.40 |  | 73 | 0 (ref) |  | 12 | 0 (ref) |  | 51 | 0 (ref) |
| Medium | 15.40 – 22.39 |  | 79 | 0.05 (-0.18; 0.28) |  | 53 | 0.40 (0.06; 0.75) |  | 32 | 0.09 (-0.14; 0.32) |
| High | ≥22.40 |  | 77 | 0.23 (-0.01; 0.46) |  | 51 | 0.62 (0.26; 0.98) |  | 11 | -0.12 (-0.49; 0.26) |
|  |  |  |  |  |  |  |  |  |  |  |
| **Diurnal glucose (mmol/L)** |  |  |  |  |  |  |  |  |  |  |
| Low | <15.40 |  | 73 | 0 (ref) |  | 12 | 0 (ref) |  | 51 | 0 (ref) |
| Medium | 15.40 – 22.39 |  | 79 | 0.09 (-0.10; 0.29) |  | 53 | 0.27 (-0.02; 0.57) |  | 32 | 0.07 (-0.15; 0.29) |
| High | ≥22.40 |  | 77 | 0.17 (-0.03; 0.37) |  | 51 | 0.37 (0.08; 0.66) |  | 11 | 0.03 (-0.36; 0.41) |

Analyses adjusted for age, sex, and body mass index. Data presented as the mean difference in outcome (with 95% confidence interval) with respect to the reference group.

| **Table A.3:** Association aspartate Aminotransaminase and measures of glycaemia in three different cohorts | | | | | | | | | | |
| --- | --- | --- | --- | --- | --- | --- | --- | --- | --- | --- |
|  | Range |  | AGO | |  | Switchbox | |  | GOTO | |
|  | U/L |  | N | Beta (95% CI) |  | N | Beta (95% CI) |  | N | Beta (95% CI) |
| **24-h mean glucose (mmol/L)** |  |  |  |  |  |  |  |  |  |  |
| Low | <23.30 |  | 73 | 0 (ref) |  | 52 | 0 (ref) |  | 42 | 0 (ref) |
| Medium | 23.30 – 27.29 |  | 77 | -0.06 (-0.25; 0.12) |  | 34 | -0.07 (-0.31; 0.16) |  | 27 | -0.07 (0.33; 0.19) |
| High | ≥27.30 |  | 79 | 0.05 (-0.13; 0.24) |  | 30 | -0.15 (-0.36; 0.07) |  | 25 | -0.17 (-0.42; 0.08) |
|  |  |  |  |  |  |  |  |  |  |  |
| **Noctornal glucose (mmol/L)** |  |  |  |  |  |  |  |  |  |  |
| Low | <23.30 |  | 73 | 0 (ref) |  | 52 | 0 (ref) |  | 42 | 0 (ref) |
| Medium | 23.30 – 27.29 |  | 77 | -0.06 (-0.28; 0.17) |  | 34 | -0.06 (-0.40; 0.27) |  | 27 | 0.05 (-0.24; 0.34) |
| High | ≥27.30 |  | 79 | 0.07 (-0.16; 0.30) |  | 30 | -0.16 (-0.44; 0.12) |  | 25 | -0.22 (-0.47; 0.03) |
|  |  |  |  |  |  |  |  |  |  |  |
| **Diurnal glucose (mmol/L)** |  |  |  |  |  |  |  |  |  |  |
| Low | <23.30 |  | 73 | 0 (ref) |  | 52 | 0 (ref) |  | 42 | 0 (ref) |
| Medium | 23.30 – 27.29 |  | 77 | -0.07 (-0.26; 0.12) |  | 34 | -0.09 (-0.31; 0.13) |  | 27 | -0.09 (-0.36; 0.18) |
| High | ≥27.30 |  | 79 | 0.04 (-0.16; 0.23) |  | 30 | -0.13 (-0.36; 0.09) |  | 25 | -0.15 (-0.42; 0.12) |

Analyses adjusted for age, sex, and body mass index. Data presented as the mean difference in outcome (with 95% confidence interval) with respect to the reference group.

| **Table A.4:** Association gamma-glutamyltransferase and measures of glycaemic variability in three different cohorts | | | | | | | | | | |
| --- | --- | --- | --- | --- | --- | --- | --- | --- | --- | --- |
|  | Range |  | AGO | |  | Switchbox | |  | GOTO | |
|  | U/L |  | N | Beta (95% CI) |  | N | Beta (95% CI) |  | N | Beta (95% CI) |
| **MAGE** |  |  |  |  |  |  |  |  |  |  |
| Low | <21.23 |  | 74 | 0 (ref) |  | 54 | 0 (ref) |  | 51 | 0 (ref) |
| Medium | 21.23 – 37.89 |  | 77 | 0.14 (-0.14; 0.42) |  | 41 | 0.08 (-0.21; 0.37) |  | 28 | 0.13 (-0.18; 0.44) |
| High | >37.90 |  | 75 | 0.19 (-0.11; 0.49) |  | 21 | -0.17 (-0.49; 0.14) |  | 15 | -0.20 (-0.53; 0.14) |
|  |  |  |  |  |  |  |  |  |  |  |
| **SD** |  |  |  |  |  |  |  |  |  |  |
| Low | <21.23 |  |  | 0 (ref) |  | 54 | 0 (ref) |  | 51 | 0 (ref) |
| Medium | 21.23 – 37.89 |  |  | 0.05 (-0.06; 0.15) |  | 41 | 0.02 (-0.08; 0.13) |  | 28 | 0.04 (-0.08; 0.16) |
| High | >37.90 |  |  | 0.05 (-0.06; 0.16) |  | 21 | -0.09 (-0.21; 0.02) |  | 15 | -0.06 (-0.19; 0.07) |
|  |  |  |  |  |  |  |  |  |  |  |
| **MODD** |  |  |  |  |  |  |  |  |  |  |
| Low | <21.23 |  | 74 | 0 (ref) |  | 54 | 0 (ref) |  | 51 | 0 (ref) |
| Medium | 21.23 – 37.89 |  | 77 | 0.06 (-0.03; 0.16) |  | 41 | 0.04 (-0.06; 0.14) |  | 28 | 0.06 (-0.06; 0.17) |
| High | >37.90 |  | 75 | 007 (-0.04; 0.17) |  | 21 | -0.06 (-0.20; 0.07) |  | 15 | -0.04 (-0.18; 0.10) |

Analyses adjusted for age, sex, and body mass index. Data presented as the mean difference in outcome (with 95% confidence interval) with respect to the reference group.

| **Table A.5:** Association alanine-aminotransferase and measures of glycaemic variability in three different cohorts | | | | | | | | | | |
| --- | --- | --- | --- | --- | --- | --- | --- | --- | --- | --- |
|  | Range |  | AGO | |  | Switchbox | |  | GOTO | |
|  | U/L |  | N | Beta (95% CI) |  | N | Beta (95% CI) |  | N | Beta (95% CI) |
| **MAGE** |  |  |  |  |  |  |  |  |  |  |
| Low | <15.40 |  | 73 | 0 (ref) |  | 12 | 0 (ref) |  | 51 | 0 (ref) |
| Medium | 15.40 – 22.39 |  | 79 | 0.04 (-0.24; 0.32) |  | 53 | -0.18 (-0.56; 0.19) |  | 32 | -0.06 (-0.39; 0.26) |
| High | ≥22.40 |  | 77 | -0.01 (-0.29; 0.28) |  | 51 | -0.27 (-0.67; 0.14) |  | 11 | -0.13 (-0.55; 0.29) |
|  |  |  |  |  |  |  |  |  |  |  |
| **SD** |  |  |  |  |  |  |  |  |  |  |
| Low | <15.40 |  | 73 | 0 (ref) |  | 12 | 0 (ref) |  | 51 | 0 (ref) |
| Medium | 15.40 – 22.39 |  | 79 | 0.02 (-0.08; 0.12) |  | 53 | -0.13 (-0.25; 0.00) |  | 32 | -0.04 (-0.15; 0.08) |
| High | ≥22.40 |  | 77 | 0.00 (-0.11; 0.10) |  | 51 | -0.15 (-0.29; -0.02) |  | 11 | -0.03 (-0.20; 0.14) |
|  |  |  |  |  |  |  |  |  |  |  |
| **MODD** |  |  |  |  |  |  |  |  |  |  |
| Low | <15.40 |  | 73 | 0 (ref) |  | 12 | 0 (ref) |  | 51 | 0 (ref) |
| Medium | 15.40 – 22.39 |  | 79 | 0.02 (-0.08; 0.11) |  | 53 | -0.06 (-0.18; 0.06) |  | 32 | -0.09 (-0.21; 0.02) |
| High | ≥22.40 |  | 77 | 0.00 (-0.10; 0.09) |  | 51 | -0.07 (-0.20; 0.06) |  | 11 | -0.09 (-0.25; 0.06) |

Analyses adjusted for age, sex, and body mass index. Data presented as the mean difference in outcome (with 95% confidence interval) with respect to the reference group.

| **Table A.6:** Association aspartate Aminotransaminase and measures of glycaemic variability in three different cohorts | | | | | | | | | | |
| --- | --- | --- | --- | --- | --- | --- | --- | --- | --- | --- |
|  | Range |  | AGO | |  | Switchbox | |  | GOTO | |
|  | U/L |  | N | Beta (95% CI) |  | N | Beta (95% CI) |  | N | Beta (95% CI) |
| **MAGE** |  |  |  |  |  |  |  |  |  |  |
| Low | <15.40 |  | 73 | 0 (ref) |  | 52 | 0 (ref) |  | 42 | 0 (ref) |
| Median | 15.40 – 22.39 |  | 79 | 0.01 (-0.26; 0.28) |  | 34 | 0.02 (-0.08; 0.13) |  | 27 | -0.24 (-0.58; 0.10) |
| High | ≥22.40 |  | 77 | 0.10 (-0.17; 0.38) |  | 30 | -0.09 (-0.21; 0.02) |  | 25 | -0.13 (-0.47; 0.21) |
|  |  |  |  |  |  |  |  |  |  |  |
| **SD** |  |  |  |  |  |  |  |  |  |  |
| Low | <15.40 |  | 73 | 0 (ref) |  | 52 | 0 (ref) |  | 42 | 0 (ref) |
| Median | 15.40 – 22.39 |  | 79 | -0.04 (-0.14; 0.06) |  | 34 | -0.07 (-0.17; 0.03) |  | 27 | -0.11 (-0.24; 0.02) |
| High | ≥22.40 |  | 77 | 0.01 (-0.09; 0.12) |  | 30 | -0.05 (-0.16; 0.07) |  | 25 | -0.06 (-0.19; 0.08) |
|  |  |  |  |  |  |  |  |  |  |  |
| **MODD** |  |  |  |  |  |  |  |  |  |  |
| Low | <15.40 |  | 73 | 0 (ref) |  | 52 | 0 (ref) |  | 42 | 0 (ref) |
| Median | 15.40 – 22.39 |  | 79 | -0.03 (-0.12; 0.06) |  | 34 | -0.04 (-0.15; 0.07) |  | 27 | -0.11 (-0.22; 0.00) |
| High | ≥22.40 |  | 77 | 0.06 (-0.04; 0.15) |  | 30 | -0.10 (-0.22; 0.03) |  | 25 | -0.15 (-0.27; -0.02) |

Analyses adjusted for age, sex, and body mass index. Data presented as the mean difference in outcome (with 95% confidence interval) with respect to the reference group.
